# Time-Varying Oscillatory Response of Burning Gel Fuel Droplets

**DOI:** 10.3390/gels9040309

**Published:** 2023-04-06

**Authors:** Janmejai Sharma, Ankur Miglani

**Affiliations:** Microfluidics and Droplet Dynamics Lab, Department of Mechanical Engineering, Indian Institute of Technology, Indore 453552, Madhya Pradesh, India

**Keywords:** droplet combustion, gel fuels, organic gellants, ethanol, hybrid rocket

## Abstract

Gel fuel droplets exhibit disruptive burning due to the rupture of their gellant shell, which causes the release of unreacted fuel vapors from the droplet interior to the flame in the form of jets. In addition to pure vaporization, this jetting allows convective transport for fuel vapors, which accelerates gas-phase mixing and is known to improve droplet burn rates. Using high-magnification and high-speed imaging, this study found that the viscoelastic gellant shell at the droplet surface evolves during the droplet’s lifetime, which causes the droplet to burst at different frequencies, thereby triggering a time-varying oscillatory jetting. In particular, the continuous wavelet spectra of the droplet diameter fluctuations show that the droplet bursting exhibits a nonmonotonic (hump-shaped) trend, where the bursting frequency first increases and then decreases to a point where the droplet stops oscillating. The changes in the shell structure are captured by tracking the temporal variation of the area of rupture sites, spatial movement of their centroid, and the degree of overlap between the rupture areas of successive cycles. During the initial period (immediately following its formation) when the shell is newly formed, it is weak and flexible, which causes it to burst at increasingly high frequencies. This is because the area at and around the rupture site becomes progressively weaker with each rupture in an already weak shell. This is shown by a high degree of overlap between the areas of successive ruptures. On the other hand, the shell flexibility during the initial period is demonstrated by a reversal in the motion of rupture site centroids. However, at later stages when the droplet has undergone multiple ruptures, the depletion of the fuel vapor causes accumulation of gellant on the shell, thus causing the shell to become strong and rigid. This thick, strong, and rigid shell suppresses droplet oscillations. Overall, this study provides a mechanistic understanding of how the gellant shell evolves during the combustion of a gel fuel droplet and causes the droplet to burst at different frequencies. This understanding can be used to devise gel fuel compositions that result in gellant shells with tailored properties, and therefore, control the jetting frequencies to tune droplet burn rates.

## 1. Introduction

Modern-day aerospace propulsion systems are scaling new heights of innovation in technology and performance. Alongside performance, there are key parameters like operational safety, reliability, ease of processing, reusability, and environmental safety [1,2,3,4,5,6] of fuel sources that are currently of prime concern. Gelled propellants are such propellants that have the potential to fulfill all the above-mentioned needs and can replace conventional solid and liquid fuels by incorporating their benefits and simultaneously negating their disadvantages. Therefore, they are considered favorable candidates for rocket and ramjet propulsion systems [6]. The commonly used liquid propellants offer ease of throttle control, but they have inherent drawbacks. A major disadvantage of the typical liquid propellants is the likelihood of leakage and spills during handling, which becomes a key concern specifically in the case of hazardous hypergolic propellants [7]. The gel propellants provide safety from spillage and leaks during handling in a manner akin to that of the solid propellants [7,8,9,10]. Moreover, the shear thinning behavior of gel fuels provides better injection and atomization that is analogous to liquid fuels [11,12,13,14]. Due to gelation, the stable suspension of metal additives can be completed in the gel fuel matrix. This can aid in improving performance characteristics, such as a specific impulse and energy density [3,6]. Interestingly, gel propellants resemble solid propellants in terms of storability and handling, but they have shown certain advantages over solid propellants. Unlike solid propellants, gel propellants are less sensitive to impact, friction, and electrostatic discharge, and thus have a decreased susceptibility to accidental ignition, deflagration, detonation, or thermal runaway [4]. The viscoelasticity of the gel propellants during the storage and the tendency to transform from solid-like to liquid-like within the delivery system eliminates the possibility of cracking the propellant structure and its consequent uncontrolled combustion [3].

Although gel propellants offer various advantageous avenues, as discussed earlier, their combustion behavior has nonetheless yet to be understood and deciphered. The combustion of gel propellants is often considered to be disruptive combustion behavior, which is accompanied by disruptive jetting events, as reported by Solomon et al. [15]. These disruptive jetting events are the jets of fuel vapors from the droplet interiors which occur aperiodically and asymmetrically throughout the droplet’s lifetime. A usual jetting event occurs sequentially as first the gellant crust/shell forms due to the phase separation of the gelling agent from its components (base fuel and solvent) in the vicinity to the droplet surface. Second, underneath the gellant shell, the trapped fuel begins to boil. Third, the inner surface layer of the gellant shell acts as nucleation sites for bubble growth, leading to an increase in pressure inside the shell. Eventually, the gellant shell ruptures, and subsequently, there is a release of internal pressure via the jetting of unreacted fuel vapor occurs. Finally, the jet travels to the flame envelope in the vicinity which causes disruptions in the flame. This mechanism of jetting has been reported in the literature by different studies conducted on organic gel-based propellants. The studies conducted by Solomon et al. [15] on multicomponent gel fuel droplets revealed that during combustion, a nonpermeable elastic layer of gellant is formed which prevents the evaporation of the fuel, which in turn causes the swelling of the droplet. This eventually leads to the jetting of unreacted fuel vapors and further shrinkage of the gel droplet. Moreover, in this study, the gel droplet diameter and time history revealed a fluctuation in the form of an increasing–decreasing trend for droplet diameters. Similarly, Mishra et al. [16,17] examined the combustion behavior of Jet A1 fuel gelled with Thixotrol ST as gellant and Xylene as the solvent and concluded that the key phenomena involved in the combustion were phase separation (gellant shell formation), bubble nucleation, and microexplosions, which occurred in a staged manner. They also reported a fluctuating trend in the volume time history of the gel droplets due to jetting. Cho et al. [18,19] utilized high-speed OH-PLIF experiments to characterize the trimodal behavior of flame-disrupting jets. They proposed that these jets occur from the same location repeatedly which can be attributed to the weakening of the gellant shell during subsequent jetting due to the viscoelastic nature. Furthermore, the combustion behavior of the unsymmetrical dimethylhydrazine (UDMH) droplets gelled with organic gellant was investigated by Feng et al. [20] under the oxidant convective environment, and these authors also reported that the combustion proceeds in four stages: (1) transient heat-up with phase separation of gel propellant components, (2) gellant-layer formation and bubble nucleation leading to an onset of microexplosions, (3) vigorous microexplosions, (4) and uniform regression of the droplet. It was observed that during stages 2 and 3, substantial jetting takes place, which also manifested in the form of fluctuating trends in the volume–time history. Research performed by Miglani et al. [21] provided key insights into the bursting and jetting behavior of ethanol-based organic gelled droplets of varying gellant concentrations. The findings of this study confirmed that due to the viscoelasticity of the gellant shells, volumetric fluctuations occur due to the oscillatory bursting of the gel droplets. They reported that the lower concentration of gellant exhibited an oscillatory bursting behavior in the form of rupture cascades that occurred in the same region of the droplet. On the contrary, for the high concentration of gellant, the ruptures took place at dispersed locations of the droplet surface.

The studies conducted by Sharma et al. [22] provide insights into the jetting dynamics and their subsequent characterization. They employed dual mirror Z-type Schlieren imaging to visualize the jetting behavior of ethanol-based organic gel fuels and found that the jetting occurs via two modes, i.e., oscillatory, and isolated. Furthermore, as per their findings, single and multiple jetting events also occur, which fall under the category of an isolated jetting mode. In this study, the jets were further classified into three categories—flame distortion, fireball, and pinhole jets—which are based on the magnitude of velocities and the degree of disruption caused by these jets in the flame envelope. Moreover, it is proposed that the nature and type of the gellant shell strongly influence the combustion behavior of the gel fuel droplets.

A survey of the relevant literature reveals that the combustion behavior of gel fuel has been analyzed either as a function of the initial droplet size and the ambient conditions [16,17] or the functional properties such as the parent fuel, the type of gellant (organic, inorganic, and cryogel) and their concentration [15,16,17,21,22,23,24,25,26]. While these studies have focused on determining droplet burn rates, a key mechanism that governs the combustion and jetting behavior in gel fuel droplets is the formation of a gellant shell, whose evolution during the droplet lifetime has been unexplored. Therefore, a key objective of this study was to investigate the temporal evolution of the gellant shell in burning ethanol-based organic gel fuel with hydroxypropyl methylcellulose (HPMC) as a gellant. By analyzing one of the modes of jetting, namely, the oscillatory jetting [21], and the extracting the key parameters such as the droplet oscillation frequency (using continuous wavelet spectra, CWT) [27], the area and overlap of rupture sites, as well as the movement of their centroid transformation in the gellant shell with time, can be inferred as being initially thin, weak, and flexible to becoming thick, strong, and rigid at later stages. 

## 2. Results

**Figure 1:**
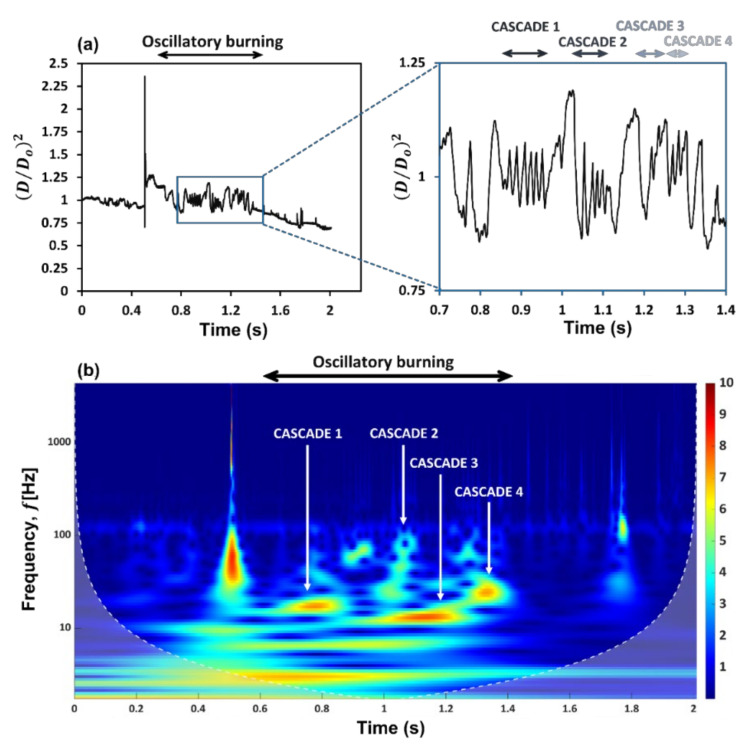
(**a**) Diameter time history of ethanol-based HPMC-3% gel fuel droplet. (**b**) Continuous wavelet spectra (CWT) or scalogram of the diameter time signal showing frequencies for diametrical fluctuations.

The droplet diameter history (Figure 1a) shows that in the disruptive burning phase, the gel fuel combustion is characterized by the occurrence of intermittent oscillations, which appear as four distinct bands. This is evident from the continuous wavelet spectra (CWT or scalogram) of the time series signal of the droplet diameter fluctuations, which shows the occurrence of four distinct frequency bands (Figure 1b). Each of these frequency bands corresponds to an oscillatory cascade which is composed of a series of rupture–recovery cycles of the gel fuel droplet. 

**Figure 2:**
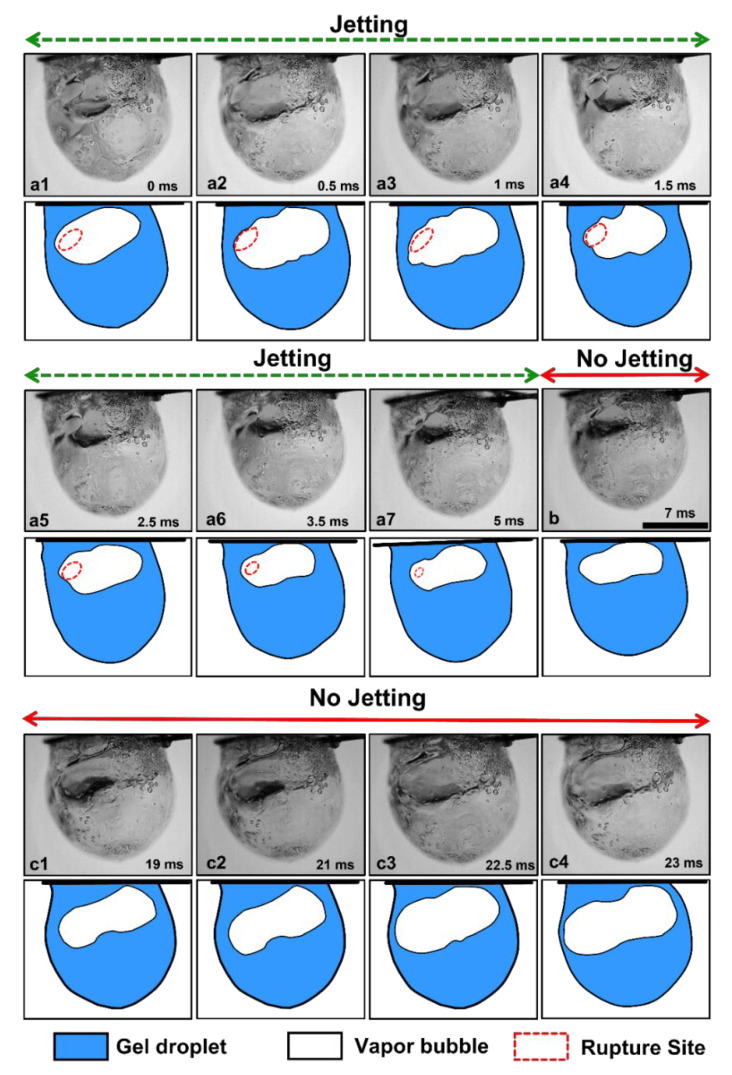
High-magnification images of a burning gel fuel droplet undergoing a single rupture–recovery cycle. The accompanying schematics are shown at the bottom of each image. (**a1**) Rupture of the gellant shell and (**a2**–**a7**) jetting of unreacted fuel vapors from the rupture site. (**b**) Recovery of the rupture site. (**c1**–**c4**) Bubble growth and droplet expansion. The time stamps are shown on each image, and the scale bar equals 1 mm.

Figure 2 shows the temporal evolution of the burning gel fuel droplet during a single rupture–recovery cycle. As seen in Figure 2, each cycle is divided into three stages: First, the gellant shell ruptures at a weak spot with the formation of a hole or site, which marks the beginning of the cycle (see Figure 2(a1)). Second, the jets of unreacted fuel vapor are released via this rupture site. This represents the time period of active jetting ($t_{a}$); see Figure 2(a2)–(a7). Third, the ruptured site recovers due to the viscoelastic nature of the shell (Figure 2b), which allows the bubbles inside the droplet to grow and dilate the droplet (Figure 2(c1)–(c4)). This in turn stresses the gellant shell to the point where it ruptures again and initiates the next rupture–recovery cycle. This implies that the complete recovery of the gellant shell, i.e., the closure of the ruptured site marks the end of the active jetting period, and therefore, serves as a prerequisite condition to initiate the next rupture–recovery cycle. In this way, each rupture–recovery cycle can be divided into two time durations, one with jetting $\left(t_{a}\right)\;$ and the other without it $\left(t_{ia}\right)$. Since the shell rupture corresponds to the efflux of unreacted fuel vapors via jetting, the jetting frequency is the same as the droplet oscillation frequency.

**Figure 3:**
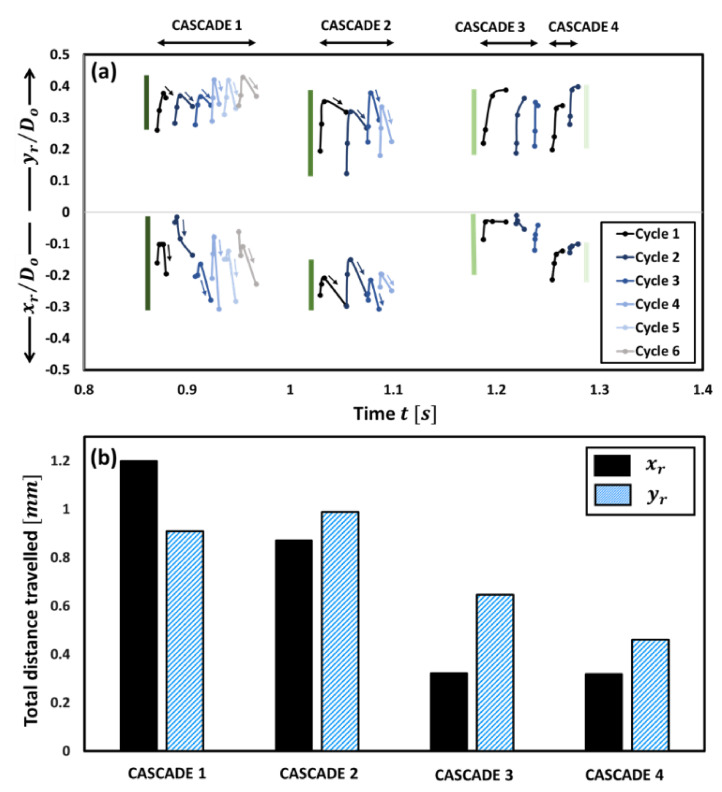
(**a**) Spatial movement of the centroid coordinates ($x_{r},\;y_{r}$) of rupture sites with time for each cascade. The double-headed arrows on the top show the time duration of each cascade. The vertical bars indicate the extent of spatial shifting. (**b**) The total distance traversed by both the centroid coordinates during each cascade.

The CWT (see Figure 1b) shows that the droplet diameter fluctuations exhibit a hump-shaped trend with time, where the oscillation frequency first increases and then decreases. Specifically, the oscillation frequencies range from ~15–18 Hz in the first band, increase by ~3–4 times to ~55–72 Hz in the second band, then decrease significantly to ~12–14 Hz in the third band, and finally, increase slightly to ~23–26 Hz in the fourth band. Note that these frequency ranges are extracted from the CWT of the droplet diameter fluctuations and are corroborated with the oscillation frequencies calculated directly from the high-speed videos as follows: fcycle=1ta+tia. The observed change in the oscillation frequency of the droplet is due to the change in the relative dominance of the two competing mechanisms over its lifetime, namely, the jetting of unreacted fuel vapors versus the gellant shell formation. The time evolution of the gellant shell can be understood based on Figure 3, where the top figure shows the spatial movement of the centroid of the rupture site xrDo,yrDo with time for each rupture–recovery cycle, while the bottom figure shows the total distance traversed by the rupture sites during each cascade.

**Figure 4:**
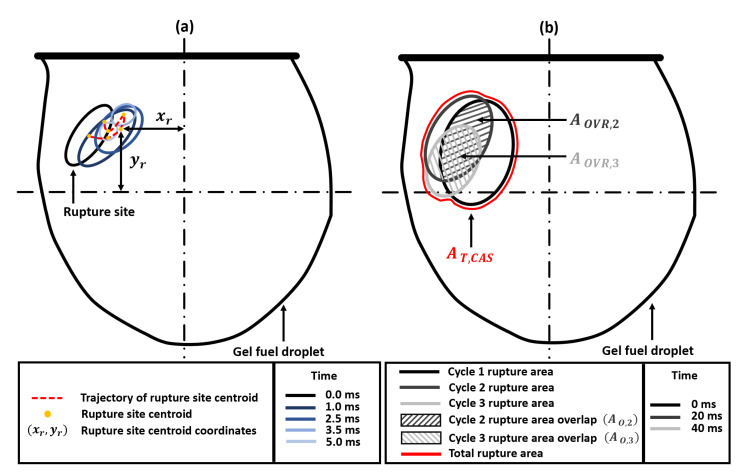
Schematic diagram showing the key geometrical parameters for analyzing the temporal evolution of the gellant shell. (**a**) Spatial movement of the rupture site centroid ($x_{r},\;y_{r}$) during a single rupture–recovery cycle for a representative case. (**b**) The maximum rupture area of each cycle, with the rupture area overlapping between successive cycles ($A_{O,2}$ and $A_{O,3}$), and the total rupture area for the cascade ($A_{T,CAS}$).

The rupture site, its centroid coordinates, and the spatial movement of these centroid coordinates for a single cycle are shown schematically in Figure 4. First, it is evident from Figure 3b that the total distance travelled by the centroids of the rupture sites is significantly more for cascades 1 and 2 compared to the following cascades 3 and 4.

**Figure 5:**
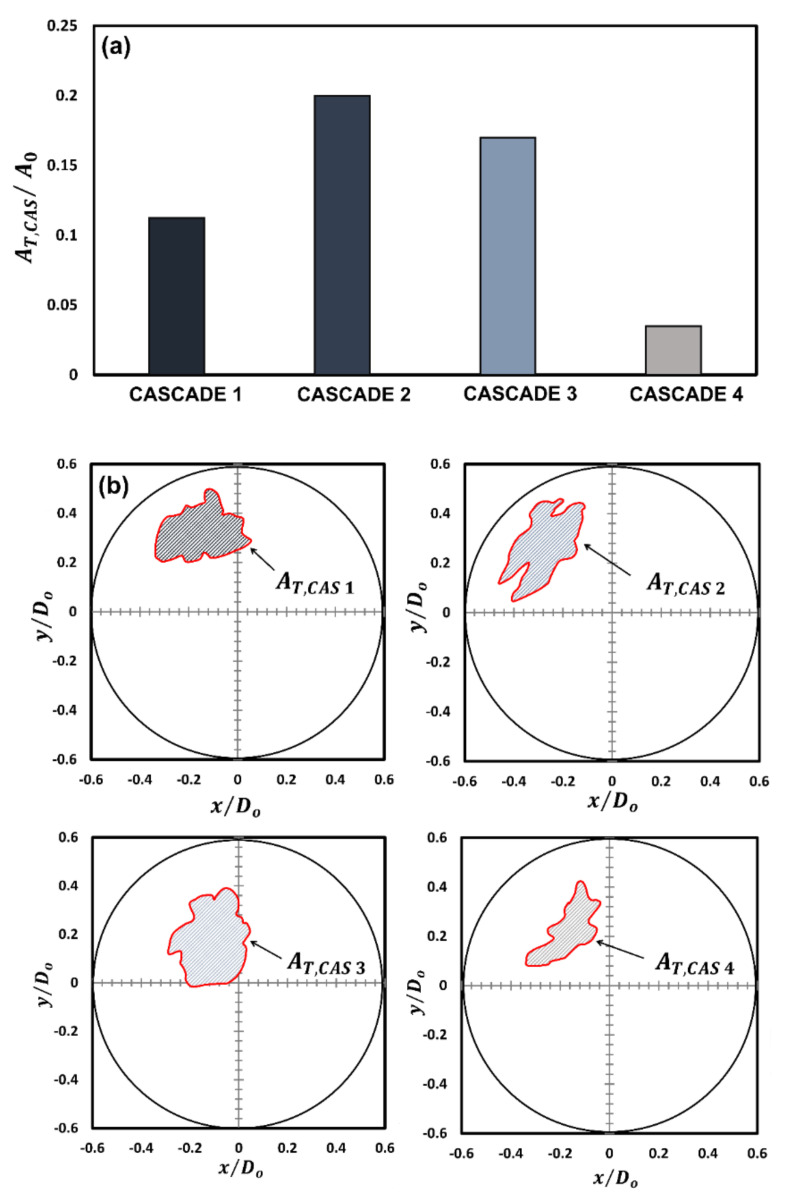
A comparison of the total rupture area for each cascade: (**a**) graphical representation and (**b**) schematic representation showing the area relative to the initial droplet area.

For instance, the distance traversed by the rupture sites in x-direction during cascades 1 and 2 is ~3 times that travelled during cascades 3 and 4, respectively, while that travelled in the y-direction is ~1.7 times compared to that travelled during cascades 3 and 4, respectively. This indicates that the gellant shell remains flexible during the first two cascades but becomes increasingly rigid with the following cascades. An important indicator of the shell flexibility during cascades 1 and 2 is the retraction or reversal in the movement of the rupture site centroids, as shown by the arrows in Figure 3a. This reversal means that the gellant shell expands as it ruptures and then contracts as the rupture site recovers. Note that the reversal of the rupture site centroid coordinates is absent in the case of cascades 3 and 4, thus indicating that the gellant shell is stiffer or more rigid during these cascades. Secondly, the total distance travelled by the centroids of rupture sites increases from cascade 1 to cascade 2 and is the highest for the latter among all cascades. This is because, with several rupture cycles occurring in cascade 1, the gellant shell becomes progressively weaker at and around the rupture site, which makes it prone to more frequent ruptures. The weakening of the rupture site can be inferred from Figure 5, which shows the total ruptured area for each cascade. From a droplet-level perspective, it is clear from Figure 5 that the shell ruptures repeatedly in the same region of the droplet (second quadrant), which becomes a weak spot, and therefore, a preferential rupture site. However, the total rupture area corresponding to each cascade increases from cascade 1 to 2 and then decreases for cascades 3 and 4, with cascade 2 having the maximum rupture area. In particular, the total rupture area of cascade 2 is ~1.8 times higher compared to cascade 1 and ~1.7 and 5.8 times higher compared to cascades 3 and 4, respectively (Figure 5a). The occurrence of maximum rupture area for cascade 2 means that a larger portion of the gellant shell becomes weaker, and therefore, the droplet naturally bursts at higher frequencies. Figure 1 shows that the droplet oscillation frequency resulting from its shell rupture and recovery is highest in cascade 2. The fact that the total rupture area reduces after cascade 2 means that the weak region of the gellant shell shrinks with progressive cascades. This is an indicator of the increasing shell stiffness, i.e., the shell becoming more rigid.

**Figure 6:**
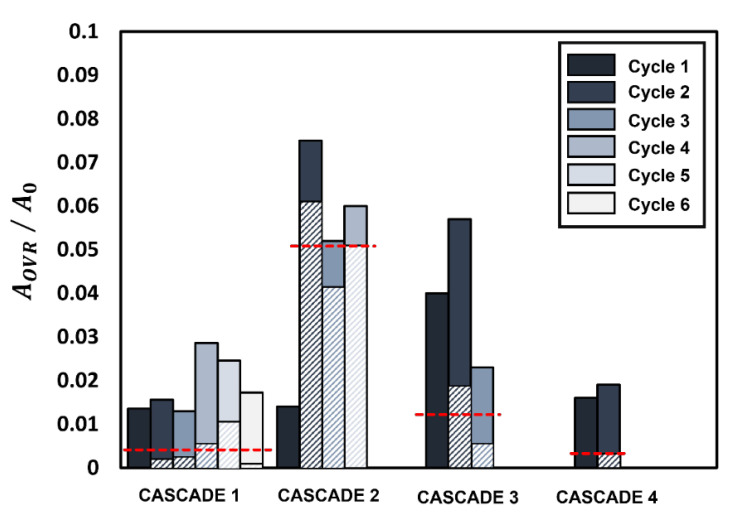
The variation of the normalized overlap area of the rupture sites ($A_{OVR}/A_{0}$) between successive cycles with each cascade. Solid bars represent the rupture site area for each cycle while shaded bars represent the percentage overlap between successive cycles. The horizontal red dotted lines represent the average overlap for each cascade.

The weak and flexible nature of the gellant shell during the initial two cascades and its strong and rigid nature during the following cascades are further confirmed by analyzing the extent of overlap between the rupture areas of successive cycles for each cascade, as shown in Figure 6. Figure 6 illustrates the normalized projected rupture area for each cycle (solid bar) and the amount of overlap of rupture area between successive cycles (hatched bar) for each cascade. The overlap area exhibits the expected hump-shaped trend, where the amount of overlap between the rupture sites areas increases from cascade 1 to 2, reaches a maximum at cascade 2, and then reduces for cascades 3 and 4. The average overlap area of rupture sites for each cascade is shown by the horizontal dotted line in Figure 6. The average overlap area for cascade 2 is ~10, 5, and 25 times higher compared to cascades 1, 3, and 4, respectively. A large degree of overlap means that the ruptures occur regularly in the same region of the gellant shell. This weakens the shell and causes it to rupture frequently, thereby increasing the droplet bursting frequency. In contrast, the low degree of overlap in cascades 3 and 4 implies that the tendency of successive ruptures to occur repeatedly at the same location declines after the second cascade. Another indicator of the gellant shell becoming increasingly rigid is the decreasing number of cycles per cascade from six cycles in cascade 1 to just two cycles in cascade 4. Furthermore, the absence of cascades after the fourth cascade indicates that by the time the droplet reaches the fourth cascade, the gellant shell becomes rigid enough that it eventually prevents the occurrence of any further oscillatory rupture–recovery cycles. Therefore, a gradually increasing rigidity of the gellant shell eventually suppresses the oscillatory burning behavior of the gel fuel droplet.

The increasing strength and rigidity of the gellant shell with time can be attributed to two processes: the efflux of unreacted fuel vapors from the droplet via jetting and the evaporation of base fuel (ethanol). Both these processes cause depletion of the base fuel from the droplet, and therefore, lead to an increase in the gellant concentration inside the droplet. This in turn leads to a gradual accumulation of the gellant on the shell, thereby increasing the shell thickness. During the initial two cascades (i.e., cascades 1 and 2), it is the efflux of unreacted fuel vapors via jetting that promotes the shell build-up process, as seen by high oscillation or jetting frequency. In contrast, during cascades 3 and 4, the jetting or the oscillation frequency is reduced due to a relatively strong and rigid shell, and therefore, it is the fuel vaporization that governs the shell build-up process. At a point where the shell becomes sufficiently rigid, it is unable to undergo the rupture–recovery oscillatory cycles. This is marked by the end of cascade 4. Note that while the oscillatory jetting terminates with cascade 4, the jetting continues in the form of individual isolated jets that occur randomly during the remaining droplet lifetime.

## 3. Conclusions

In general, the results presented in Figure 1, Figure 2, Figure 3, Figure 4, Figure 5 and Figure 6 demonstrate a time-varying oscillatory response in the combustion of an organic gel fuel droplet with ethanol as the base fuel and hydroxypropyl methylcellulose (HPMC). This study provides a phenomenological understanding of the evolution of the gellant shell during the combustion of gel fuel droplets that burst at different frequencies. Furthermore, these insights can be incorporated to formulate gel fuel compositions that result in gellant shells with customized properties. Consequently, controlling and altering the jetting frequencies can enhance droplet burn rates. The following are the conclusions that can be drawn from this study:

- The high-magnification and high-speed imaging of the gel droplet combustion reveals that the viscoelastic gellant shell at the droplet surface evolves during the droplet’s lifetime, which leads to the bursting of gel fuel droplet at different frequencies, thereby triggering a time-varying oscillatory jetting in the form four rupture cascades. In each of these cascades, it is observed that the number of rupture cycles differs and has a decreasing trend. The highest number of rupture cycles is found in cascade 1 with 6 cycles, while the lowest is in cascade 4 with 2 cycles. Furthermore, in cascades 2 and 3, the number of rupture cycles is 4 and 3, respectively.
- The continuous wavelet spectra of the droplet diameter fluctuations elucidate the droplet bursting trait which exhibits a nonmonotonic (hump-shaped) trend. Indeed, the bursting frequency first increases from cascade 1 (15–18 Hz) to cascade 2 (55–72 Hz) and then decreases in cascade 3 (12–14 Hz) and cascade 4 (23–26 Hz), respectively to an extent at which the droplet stops oscillating.
- The assessment of variations in the gellant shell (from thin-flexible to thick-strong and rigid) during the combustion was captured by tracking the temporal variation of the area of rupture sites, the spatial movement of their centroid, and the degree of overlap between the rupture areas of successive cycles for each cascade.
- The newly formed gellant shell during the initial period (immediately after its formation), is weak and flexible; consequently, bursting can be observed at increasingly high frequencies. This is because the area at and in the vicinity of the rupture site becomes progressively weaker with each ensuing rupture in an already weakened shell. This was indicated by a high degree of overlap between the areas of successive ruptures, which was found to be highest in cascade 2 (~81%).
- The shell flexibility during cascades 1 and 2 exhibited a retraction and reversal in the motion of rupture site centroids. However, in the ensuing cascades 3 and 4, when the droplet had undergone multiple ruptures, this retraction and reversal of the rupture site vanished. Consequently, the depletion of the fuel vapor aids in the accumulation of gellant on the shell, thus leading to the formation of a thick, strong, and rigid shell, which suppresses the droplet oscillations.

## 4. Materials and Methods

### 4.1. Materials and Fuel Formulation

The gel fuel formulated in the study comprises a tricomponent system of research-grade ethanol (99.8% pure; CAS No. 64-17-5) as the base fuel, hydroxypropyl methylcellulose (HPMC; CAS No. 9004-65-3, bulk density $\rho_{b}$~689.19 kg/m^3^) as the organic gellant, and deionized water as the HPMC, consisting of both hydroxyl and methoxy groups ranging between 7% and 12% and between 28% and 30%, respectively [28,29]. All these components were procured from Sigma-Aldrich Co. The yield stress value and composition of HPMC-based [21] test fuel is given in Table 1.

This gel fuel is prepared in a manner such that in the formulation, the amount of base fuel (i.e., ethanol) is maximized while the phase stability is maintained. The fuel is formulated in the following three steps: First, the ethanol is added to the organic gellant (HPMC) and stirred manually for ~2 to 3 min. Second, the solvent, i.e., deionized water is added to the ethanol–gellant mixture for gelation and subjected to mechanical stirring at 500 rpm by using a three-blade impeller for ~2 min. Third, the resulting gel is left undisturbed for ~2 days under ambient conditions. This serves the purpose of enabling the formation of a stable 3D gel matrix. During this duration, gels are observed for phase separation that may occur during the gelation.

### 4.2. Experimental Facility and Image Acquisition

**Figure 7:**
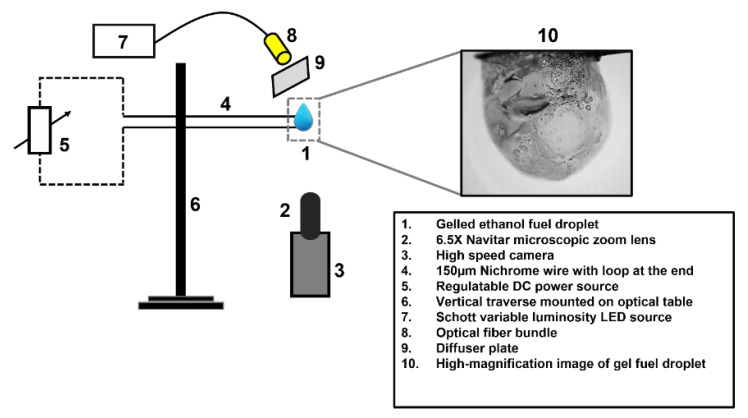
The schematic diagram of the experimental test facility for capturing the oscillatory combustion behavior of the ethanol-gel fuel droplets.

The experimental test facility used for investigating the oscillatory combustion behavior of ethanol-gel fuel droplets in the pendant mode at ambient conditions and under normal gravity is shown schematically in Figure 7. In this configuration, a 2.8 µL gel fuel drop is dispensed by utilizing a volume-calibrated microliter syringe. The gel fuel droplet is suspended over a fused quartz wire of an approximate size of 80 µm. The small diameter of the quartz wire ensures that it offers negligible physical and thermal interference during combustion due to its small size and low thermal conductivity (1.4 W/m-K at 293 K), respectively. The droplet is ignited using a 150 µm nichrome wire which is powered via a DC power supply. To capture the oscillations dynamics of the gel fuel droplet undergoing combustion, an ultra-high-speed PHOTRON FASTCAM SA-X2 (Photron, Daejeon, Republic of Korea) coupled with a 6.5× Navitar Zoom lens is used. The oscillation dynamics are recorded at 10,000 fps with an exposure time of 60 µs and a spatial resolution of 3.9 µm/pixel [30,31,32]. By using HED, the initial diameter of the gel fuel droplets was determined to be 1.65 ± 0.1 mm with an error of ±3%.

### 4.3. Data Reduction

Since gel fuels are non-Newtonian, it is difficult to dispense a perfect spherical-shaped gel droplet, and therefore, the projected area-based equivalent diameter is used to determine the droplet diameter fluctuations with time. The HED algorithm-based image processing captures all the four parameters accurately namely, the area of rupture sites, centroid of rupture sites, the spatial movement of rupture sites, and the percentage overlap between successive rupture areas. The projected-area diameter is determined using an in-house developed deep learning-based holistically nested edge detection (HED) algorithm, as reported previously [33,34,35,36]. HED enables automated learning of multiscale and multilevel features in a droplet image and reconstructs a continuous droplet. Furthermore, the extracted droplet shape is verified by recreating the droplet boundary by taking a polar plot of the distances from the centroid to the droplet edge based on their angle. This is carried out by feeding an array of computed radial distances and their corresponding angles to the standard polar plot function from the matplotlib python library. Based on the edge detection, the maximum error in determining the droplet diameter is 3% [33]. The temporal evolution of the droplet oscillation frequencies is determined via the continuous wavelet spectra (CWT) of the time series signal of droplet diameter fluctuations in-built into the MATLAB R2021b version software. The continuous wavelet spectra (CWT) or the scalogram represent the frequency-time domain analyses, where the analyzing function is a wavelet, ψ. The CWT compares the time series signal to the versions of a wavelet that is shifted in time and compressed or stretched, i.e., scaled. By comparing the signal to the wavelet at various scales and positions, a function of two variables is generated. It has two implications, if the wavelet is complex-valued, the CWT is a complex-valued function of scale and position. If the signal is real-valued, the CWT is a real-valued function of scale and position. (“Continuous Wavelet Transform and Scale-Based Analysis”) For a scale parameter, *a* > 0 and the position, *b*, the CWT is given as follows:

$$C\left[a,b;f\left(t\right)\psi \left(t\right)\right]=\int_{-\infty }^{\infty }f\left(t\right)\frac{1}{a}\psi^{*}\left(\frac{t-b}{a}\right)dt$$

where * denotes the complex conjugate. By continuously varying the values of the scale parameter a and position parameter b, the CWT coefficients $C\left(a,b\right)$ are obtained, and multiplying each coefficient by the appropriately scaled and shifted wavelet yields the constituent wavelets of the original signal. In the CWT analysis, scale and frequency have a crucial relationship. A smaller scale leads to compression of the wavelet function, and hence a higher frequency resolution is obtained but a low time resolution is subsequently observed. On the contrary, a longer scale *a* lead to the stretching of the wavelet function, which results in a low-frequency resolution but high time resolution. In the CWT spectral distribution shown in Figure 1, the white line represents the cone of influence, below which the area in the scalogram is affected by edge-effect artifacts, and therefore, treated as unreliable. These effects in the scalogram arise from areas where the stretched wavelets extend beyond the edges of the observation interval. Above the white line, the information provided by the scalogram is an accurate time-frequency representation of the data. In this study, the morse wavelet is used for generating the scalogram of the time-varying droplet diameter fluctuations.

## Figures and Tables

**Table 1:** The composition of organic gellant-based ethanol gel fuel.

Gellant HPMC (wt. %)	Gellant Solvent Deionized Water (wt. %)	Base Fuel Ethanol wt.%)	Yield Stress (Pa)
3	15	82	23.23 ± 2.62

## Data Availability

Not applicable.
